# The Influence of Successful Treatment of Stress Urinary Incontinence and Pelvic Organ Prolapse on Depression, Anxiety, and Insomnia—A Prospective Intervention Impact Assessment Study

**DOI:** 10.3390/jcm13061528

**Published:** 2024-03-07

**Authors:** Urszula Kalata, Michał Jarkiewicz, Andrzej Pomian, Aneta Janina Zwierzchowska, Edyta Horosz, Wojciech Majkusiak, Beata Rutkowska, Ewa Monika Barcz

**Affiliations:** 1 Chair of Gynaecology and Obstetrics, Medical Faculty Collegium Medicum, University of Cardinal Stefan Wyszynski, Bursztynowa 2 Str., 01-938 Warsaw, Poland; u.kalata@uksw.edu.pl (U.K.); a.pomian@uksw.edu.pl (A.P.); a.zwierzchowska@uksw.edu.pl (A.J.Z.); e.horosz@uksw.edu.pl (E.H.); w.majkusiak@uksw.edu.pl (W.M.); b.rutkowska@uksw.edu.pl (B.R.); 2 3rd Department of Psychiatry, Institute of Psychiatry and Neurology, 02-957 Warsaw, Poland; m.jarkiewicz@ipin.edu.pl

**Keywords:** stress urinary incontinence, pelvic organ prolapse, depression, anxiety, insomnia, surgery

## Abstract

**Introduction**: The association between pelvic floor disorders (PFDs) and psychiatric conditions is an area of emerging interest. The causal direction of this relationship, however, remains ambiguous; it is unclear whether PFDs directly contribute to the deterioration of mental health or if pre-existing psychiatric conditions such as depression exacerbate the symptoms of PFDs. This study aimed to evaluate the effects of successful surgical treatment for stress urinary incontinence (SUI) and pelvic organ prolapse (POP) on symptoms of depression, anxiety, and insomnia. **Materials and Methods**: This investigation focused on patients who underwent successful surgical interventions for SUI and POP. Both subjective and objective symptoms of PFDs, along with psychiatric status, were assessed before and after the surgical procedures. **Results**: This study found that successful surgical treatment of SUI and POP led to a significant reduction in anxiety scores. Additionally, in patients with SUI, successful treatment was objectively associated with a decrease in the severity of insomnia. Alleviation of symptoms associated with the lower urinary tract, prolapse, and colorectal–anal region following POP surgery was correlated with improvements in depression and anxiety but not insomnia. Subjectively assessed improvements in SUI subjective symptoms were linked to reductions in the severity of depression, anxiety, and insomnia in patients who underwent anti-incontinence surgery. **Conclusions**: These findings suggest a potential cause-and-effect relationship between PFDs and certain psychiatric disorders, highlighting the importance of successful treatment of PFDs in mitigating symptoms of depression, anxiety, and insomnia.

## 1. Introduction

Pelvic floor disorders (PFDs), including conditions such as stress urinary incontinence (SUI) and pelvic organ prolapse (POP), represent significant clinical and societal challenges [1]. These disorders affect millions of women worldwide, profoundly impacting their quality of life in various aspects. Symptoms associated with PFDs include lower urinary tract issues, colorectal problems, sexual dysfunctions, and a reduction in self-esteem [2]. Moreover, recent research has highlighted a concerning link between PFDs and the emergence of psychiatric disorders. Particularly, depression, anxiety, and sleep disturbances are among the most prevalent mental health issues observed in individuals suffering from these conditions. While the majority of studies have focused on the mental health implications of overactive bladder syndrome, especially symptoms of urgency and frequency, the influence of SUI and POP on mental health is also garnering increased attention and analysis.

Among the notable research contributions to this field, one particularly compelling study focused on the correlation between PFDs and depressive disorders. In a meticulously conducted case–control investigation, Mazi et al. demonstrated that among a cohort of one hundred patients diagnosed with PFDs, the prevalence of depressive symptoms was found to be threefold higher compared to a matched control group devoid of PFDs. Furthermore, it was statistically evident that the quality of life reported by patients afflicted with PFDs was significantly inferior when contrasted with that of individuals without such disorders. However, a limitation of this study was its broad categorization of PFDs, encompassing both urinary and fecal incontinence as well as pelvic organ prolapse, without delving into the specific impacts of each pathology. Despite this limitation, the study unequivocally highlighted the detrimental effects of PFDs on mental health, underscoring the pressing need for comprehensive clinical attention to these issues, and it provided important data on their impact on the mental state of patients [3].

In their research, Zuluaga et al. identified a significant correlation between the presence and intensity of overactive bladder syndrome (OABs) and SUI, with additional mental health issues, notably anxiety, and, more specifically, the association between nocturia and this particular symptom. Furthermore, the study highlighted a greater prevalence of depression linked with SUI and sensations of incomplete urinary bladder emptying [4]. In the context of POP, individuals were found to exhibit moderate to severe levels of catastrophizing. Moreover, psychological assessments of depression and anxiety, utilizing the Kessler Psychological Distress Scale (K-10), demonstrated a connection with the extent of bother caused by POP. It is crucial to acknowledge that this observed impact on mental health appears to be directly related to the severity of subjective symptoms experienced by the patients rather than objective clinical signs, suggesting that the subjective experiences of individuals with POP play a pivotal role in the deterioration of their mental well-being [5]. 

The observations of Swenson and colleagues regarding the postpartum period are also very interesting and clinically important. The authors showed that urinary incontinence during pregnancy and persistent pelvic pain are independent risk factors for postpartum depression and that these problems may be an independent cause of mental deterioration, regardless of the period of life in which they appear. Based on the data obtained, it is believed that patients with urinary incontinence should be particularly monitored for the occurrence of depressive disorders [6]. 

The national population-based cohort study conducted in Taiwan uncovered a bidirectional relationship between anxiety, depression, and lower urinary tract symptoms (LUTS). When adjustments were made for variables such as age, gender, and existing medical comorbidities, it was found that individuals with LUTS were at a significantly increased risk—2.12 times more likely for anxiety and 2.03 times more likely for depression—of developing these mental health conditions. Conversely, those diagnosed with anxiety and depression were also more prone to experiencing LUTS, with probabilities standing at 2.01 and 2.37 times higher, respectively. Furthermore, the researchers highlighted a critical consideration: the actual prevalence of psychiatric disorders among this population might be underreported. This potential underestimation arises because the study included only those individuals who sought healthcare services, suggesting that the true impact of LUTS on mental health could be even more substantial [7]. This insight emphasizes the complex interplay between physical symptoms and mental health, underscoring the necessity for healthcare providers to adopt a holistic approach in the diagnosis and treatment of patients presenting with LUTS to adequately address both the physical and psychological dimensions of patient care.

In research performed by our group, it was demonstrated that not only OABs but also SUI and POP are linked to depressive disorders, anxiety, and insomnia. Our findings indicate that individuals diagnosed with PFDs exhibit higher levels of severity in these symptoms, as quantitatively assessed using the Center for Epidemiological Studies Depression Scale (CESD), the State-Trait Anxiety Inventory (STAI), and the Insomnia Severity Index (ISI), compared to individuals without pelvic floor pathology. Drawing a parallel to the findings in women during the postpartum period, our study suggests that patients suffering from SUI and POP might also benefit from psychiatric evaluation and care [8].

Understanding whether the measurable severity of conditions, such as the volume of urine leakage and the stage of POP, directly impacts the levels of anxiety, depression, and insomnia or whether specific factors associated with these disorders, e.g., voiding difficulties or urgency–frequency, play a more significant role, is crucial. In the study referenced, no direct correlation was established between the objective measures of severity for SUI and POP—specifically, the results of the 1 h pad test and POPQ scale—and the scores on the State-Trait Anxiety Inventory (STAI), Insomnia Severity Index (ISI), and Center for Epidemiological Studies Depression Scale (CES-D) among affected individuals. Conversely, a notable correlation was observed between these mental health aspects, namely depression, anxiety, and insomnia, and the subjective assessment of the quality of life related to urinary incontinence, as measured by the IIQ7 (Incontinence Impact Questionnaire). For individuals with POP, the perceived decline in quality of life across various domains, including symptoms related to the lower urinary tract, colorectal issues, and prolapse, was linked to the manifestation of symptoms of anxiety, depression, and insomnia. This suggests that, beyond the objective severity of the PFDs, the individual’s subjective experience plays a pivotal role in shaping their mental health and overall perception of well-being.

The prevalence of psychiatric disorders among patients with pelvic floor diseases, including OABs, SUI, and POP, appears to be higher, a notion that is widely supported by the body of research. However, the precise nature of the interaction between these conditions remains a subject of debate. As stated earlier, a critical question that emerges is whether PFDs directly contribute to deteriorations in mental health or whether individuals suffering from depression or anxiety might perceive the symptoms associated with these conditions as more severe, thereby negatively impacting their quality of life. Some researchers propose that depression can intensify the symptoms of POP beyond what would be expected based on the objective severity of the prolapse in individuals without depression [9]. However, a definitive understanding of the reciprocal influence between PFDs and mental health issues such as depression, anxiety, or sleep disorders would require an analysis of psychiatric symptoms following the resolution of the underlying pelvic floor condition. This approach would help clarify whether the presence of PFDs exacerbates mental health issues or, conversely, if pre-existing psychiatric conditions amplify the perceived severity of PFD symptoms, highlighting the complex interplay between physical health conditions and mental well-being.

In our earlier research, we conducted a comparative analysis between healthy women and those afflicted with SUI and POP, finding a higher prevalence of symptoms related to depression, anxiety, and insomnia among the latter group. However, the question of whether PFDs directly cause these mental health conditions remained unanswered. 

With this context in mind, the primary objective of our current study was to evaluate the effects of successful surgical interventions for SUI and POP on the severity of depression, anxiety, and insomnia symptoms. We hypothesized that if the resolution of PFDs via surgery leads to an improvement in the mental health of patients, it would bring us closer to establishing a causal link between these physical and mental health issues. This approach aims to clarify the relationship between the presence of PFDs and subsequent mental health challenges, offering insights into whether treating the physical symptoms of PFDs can positively impact psychological well-being.

## 2. Materials and Methods

The study was conducted at a university-affiliated urogynecological center, focusing on patients who were candidates for surgical intervention due to PFDs. The average duration of postoperative follow-up was nine months.

Participants were categorized into two groups:

Group 1 (POP): patients experiencing symptomatic pelvic organ prolapse (POPQ > 2) who underwent successful surgical treatment a. before and b. after the procedure.

Group 2 (SUI): patients suffering from stress urinary incontinence evaluated a. before and b. after undergoing successful surgery.

The objective criterion for successful POP treatment was defined as a POPQ scale score below 2 in all compartments. 

Beyond these objective indicators, patient perspectives on treatment were determined using validated questionnaires: the Colorectal–Anal Distress Inventory 8 (CRADI-8), the Pelvic Organ Prolapse Distress Inventory 6 (POPDI-6), and the Urogenital Distress Inventory 6 (UDI-6).

CRADI-8 focuses specifically on colorectal–anal symptoms to assess the distress they cause to patients. This tool is used to evaluate symptoms related to colorectal and anal distress, including aspects of obstructive bowel symptoms, fecal incontinence, irritative symptoms, and rectal prolapse. It aims to quantify the impact of these symptoms on a patient’s quality of life.

The POPDI-6 is also a questionnaire designed to measure the distress and impact on quality of life caused by symptoms of POP. It focuses on the experiences of pressure or heaviness in the lower abdomen, visible or palpable vaginal bulge, the necessity of manual pressure to facilitate rectal emptying, feelings of incomplete bladder emptying, and the need to manually reposition the prolapse for urination. 

The UDI-6 questionnaire assesses the impact of symptoms potentially related to prolapse, such as frequent urination, urgency, involuntary urine loss during activities, dribbling, difficulty emptying the bladder, and discomfort in the pelvic or vulvar area, to determine their level of bother to individuals.

For SUI, objective success was determined by a negative 1 h pad test (<2 g), the absence of de novo OABs, no post-void urinary retention, the absence of pelvic pain syndrome, and no exposure of the sling in the vagina. 

The subjective effect was assessed via significant quality-of-life improvements via the Incontinence Impact Questionnaire (IIQ-7). The IIQ-7 is designed to assess the impact of urinary incontinence on quality of life. 

The questionnaire assesses the impact of urinary incontinence on several critical aspects of life, including physical activities, the ability to travel, and the health of social and personal relationships. It also evaluates the emotional consequences of incontinence, such as feelings of nervousness, frustration, and depression. This approach ensures a comprehensive understanding of how incontinence affects both the physical and psychological well-being of individuals. 

Epidemiological data were collected (age, parity, number of vaginal deliveries, number of cesarean sections, age of first and last delivery, BMI, duration of symptoms, and co-morbidities) in each patient.

The study was conducted between 2021 and 2023.

After giving their written consent, all patients underwent urogynecology examination by five highly experienced gynecologists. In patients with POP, the stage of prolapse in the POPQ scale was evaluated as well, and POPDI 6, UDI 6, and CRADI-8 questionnaires were filled in before and 9 months after surgery. All patients also completed questionnaires evaluating depression (CESD Centre for Epidemiologic Studies Depression Scale, the questionnaire that pertains to an individual’s emotional and mental state, specifically relating to mood, appetite, social interactions, sleep patterns, self-esteem, and motivation); anxiety (STAI State-Trait Anxiety Inventory, the questionnaire that concerns feelings of safety, nervousness, tension, self-perception of failure, fatigue combined with agitation, and a sense of being shaken or tremulous); and insomnia (ISI—Insomnia Severity Index), the questionnaire that addresses issues related to sleep, inquiring about difficulties with falling asleep, satisfaction with current sleep quality, the impact of sleep disturbances on daily functioning such as fatigue, work performance, daily activities, concentration, memory, mood, etc. It also seeks to understand the individual’s concerns about their sleep disorders and how perceptible they believe these issues are to others in terms of affecting their quality of life) before and 9 months after surgery. In cases with SUI, results of the 1 h pad test and IIQ-7 questionnaires were collected before and 9 months after surgery.

All the questionnaires used were in Polish and were validated instruments.

The change in mental state was assessed as the difference between the scores obtained in the ISI, CESD, and STAI questionnaires before and after the surgery.

In the POP group, different techniques such as native tissue repair (in anterior or/and posterior compartment using the fascia plication method), laparoscopic sacropexy, or transvaginal mesh (Ingynious anterior, A.M.I., Feldkirch, Austria) were used according to the prolapse type, objective severity of prolapse, patient preferences, and overall health condition. In patients with SUI, the insertion of a retropubic suburethral sling was performed in all patients (bottom-top, TVT Blue Gynacare, Cincinnati, OH, USA).

The exclusion criterion was the failure to achieve a satisfactory result from the surgery (success criteria described above) and the coexistence of various pelvic floor disorders (PFDs) in a single patient.

The study was approved by the local Ethical Committee in the Medical Chamber in Warsaw (KB/1264).

Descriptive statistical analysis expressing the quantitative and categorical variables was performed with the use of R version 4.3.0 software. Normality was tested using the Lillefors and Shapiro–Wilk tests. We associated the degree and type of non-adherence using the U Mann–Whitney test. The Pearson correlation test was used to determine the correlation between quantitative variables. *p*-value < 0.05 was considered statistically significant. 

## 3. Results

In total, 348 patients were enrolled in the study. 

In the POP group (Group 1), 187 patients were enrolled. Of these, 83 patients underwent laparoscopic promontofixation, 76 native tissue repair, and 28 transvaginal mesh surgery. Characteristics of the POP group are shown in Table 1. 

The SUI group (Group 2) consisted of 161 patients who underwent retropubic sling surgery. The characteristics of the SUI group are shown in Table 2.

The most common co-morbidities were hypertension, hypothyroidism, and type II diabetes, with no significant differences between the groups. 

To assess the impact of treatment of PFDs on the mental state of patients, first, we analyzed the differences in the results of questionnaires relating to depression, anxiety, and insomnia after successful treatment of POP.

We showed a high statistical difference in anxiety scores (STAI) after successful POP surgery. 

In the case of depression scores (CESD) analysis, there was no improvement after the surgery. 

Regarding insomnia, as measured by the ISI, the analysis demonstrated a minor difference, although it was not statistically significant (Table 3). 

In the SUI group, significant differences in anxiety symptoms after surgery (STAI) were shown. 

Similar results were obtained when insomnia was analyzed before and after TVT. In the case of depression (CESD), an improvement was observed, but it was not statistically significant (Table 4).

To examine if specific psychiatric symptoms are linked to certain subjective complaints related to PFDs, an analysis was undertaken of the relationship between the elimination of particular groups of ailments and the improvement in the mental state. For this, we analyzed the connections between alterations in psychiatric conditions, as measured by the CESD, ISI, and STAI questionnaires, and changes in symptoms of PFDs, as captured by specific quality-of-life assessments (UDI-6, CRADI-8, and POPDI-6).

**Figure 1:**
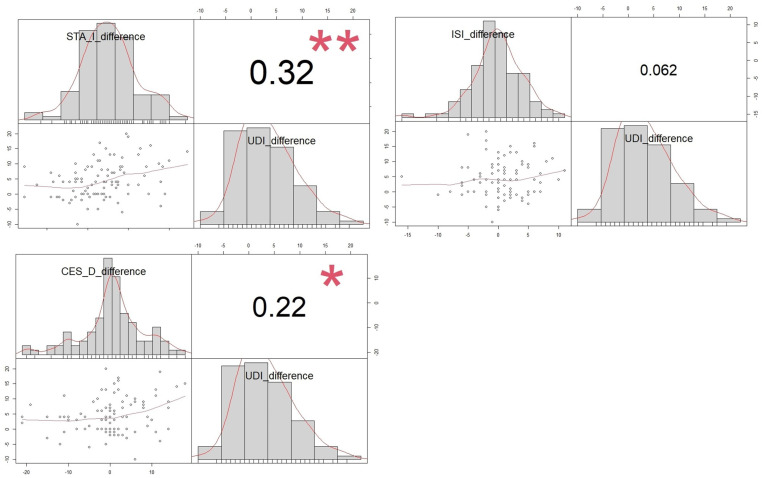
Correlation between changes in UDI 6 questionnaire and improvement in depression, anxiety, and insomnia symptoms after successful POP surgery. Correlation between ISI, STAI, CESD, and UDI-6 improvement after pelvic floor surgery is shown via a scatterplot, with each pair’s distribution, correlation coefficient, and significance levels marked (* *p* < 0.05, ** *p* < 0.01).

We showed a statistically significant correlation between the change in UDI-6 symptoms and the change in depression and anxiety scales 9 months after successful POP surgery. However, improvement in lower urinary tract symptoms (feeling of heaviness in the lower abdomen, difficulty in micturition, urinary frequency, etc.) did not improve insomnia (Figure 1).

**Figure 2:**
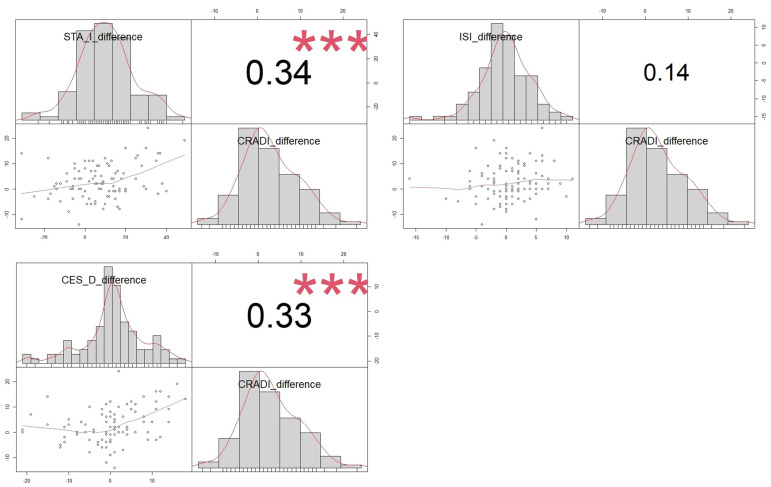
Correlation between changes in CRADI 8 questionnaire and improvement in depression, anxiety, and insomnia symptoms after successful POP surgery. Correlation between ISI, STAI, CESD, and CRADI-8 improvement after pelvic floor surgery is shown via a scatterplot, with each pair’s distribution, correlation coefficient, and significance levels marked (*** *p* < 0.001).

Changes in colorectal symptoms, as measured by the CRADI-8, show a correlation with improvements in both depression and anxiety following surgery. However, for lower gastrointestinal symptoms, enhancements in these areas did not correlate with improvements in insomnia, as illustrated in Figure 2.

**Figure 3:**
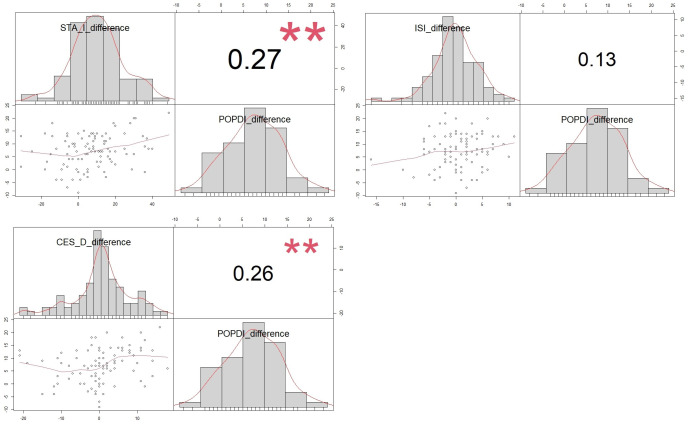
Correlation between changes in POPDI 6 questionnaire and improvement in depression, anxiety, and insomnia symptoms after successful POP surgery. Correlation between ISI, STAI, CESD, and POPDI-6 improvement after pelvic floor surgery is shown via a scatterplot, with each pair’s distribution, correlation coefficient, and significance levels marked (** *p* < 0.01).

An improvement in symptoms related to POP, as indicated by the POPDI-6, was linked to improvements on the STAI and CESD scales. However, a reduction in prolapse symptoms did not show a correlation with improvements in insomnia post-surgery, as depicted in Figure 3.

**Figure 4:**
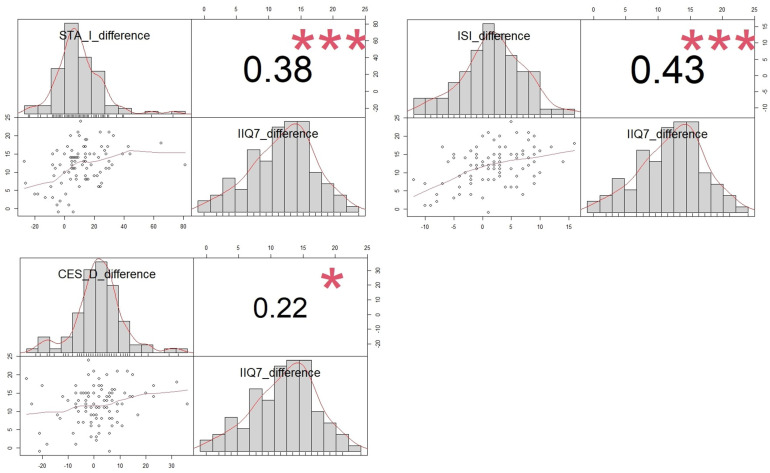
Correlation between changes in IIQ7 questionnaire and improvement in depression, anxiety, and insomnia symptoms after successful SUI surgery. Correlation between ISI, STAI, CESD, and IIQ-7 improvement post-TVT surgery is shown via a scatterplot, with each pair’s distribution, correlation coefficient, and significance levels marked (* *p* < 0.05, *** *p* < 0.001).

In patients with SUI, improvements noted in the IIQ7 questionnaire correlated positively with enhancements in all three areas of mental health, depression, anxiety, and insomnia scales, highlighting a broad impact on overall psychological well-being (Figure 4).

## 4. Discussion

PFDs negatively affect many aspects of the patient’s quality of life [10,11,12]. At the same time, it has been shown that surgical treatment of POP and urinary incontinence using different surgical methods improves patients’ quality of life in many aspects, especially as lower urinary tract, prolapse, and colorectal symptoms were concerned. Some authors show that the mean postoperative PFDI-20 (Pelvic Floor Disability Index) and PFIQ-7 (Pelvic Floor Impact Questionnaire) scores decreased by 67.50% and 76.98% postoperatively [13].

Recent research has highlighted the detrimental effects of overactive bladder syndrome (OABs) on patients’ mental health. Similarly, connections between stress urinary incontinence (SUI), pelvic organ prolapse (POP), and mental health issues like depression, insomnia, and anxiety have been identified, albeit less frequently. Studies have specifically found that urgency scores in patients with OABs are positively correlated with concurrent levels of anxiety, depression, and stress, illustrating a significant link between urinary symptoms and mental health outcomes [14].

While there is a consensus among researchers about the link between PFDs and the worsening of psychiatric conditions, the complex interplay between these health issues remains largely unexplored.

As previously noted, some researchers examining the link between PFDs and mental health suggest that pre-existing depression may intensify the subjective experience of conditions like POP or worsen reported symptoms [15]. However, there exist findings that indicate that pre-existing anxiety does not adversely impact the subjective outcomes of surgical treatments for patients with PFDs [16], highlighting a nuanced understanding of how mental health conditions influence the perception and treatment efficacy of PFDs.

The uncertainties highlighted above prompted the initiation of our present study. Within our cohort and setting, we have demonstrated that the successful treatment of pelvic organ prolapse (POP) notably reduces anxiety levels while leaving symptoms of insomnia and depression unaffected. This suggests that POP may have been the primary driver of anxiety among our patients, and its resolution led to a significant reduction in this symptom. Our findings are consistent with the findings of Larouche et al., who reported a substantial median change in preoperative to postoperative scores on the Beck Anxiety Inventory. However, they noted that postoperative pelvic floor symptoms, particularly those associated with pain or other complications, were linked to persistent postoperative depressive symptoms [17]. Nonetheless, our study’s scope precludes us from discussing the impact of complications on the presence of depression or anxiety, given that we exclusively included uncomplicated patients after successful treatment.

Our investigation revealed that successful surgery for SUI led to a reduction in anxiety and insomnia symptoms, with no notable impact on depression scales. Similar findings were reported by other researchers, who observed a favorable effect of SUI treatment on anxiety symptoms and, in some instances, depression, following various surgical interventions compared to conservative approaches. While our patient cohort shares similarities with those in the cited studies, it is worth noting that their research was conducted on a notably smaller scale. Additionally, the mentioned articles did not address insomnia and were based on significantly smaller sample sizes [18]. Another study focusing solely on sling surgery demonstrated postoperative improvements in anxiety, similar to our findings, as well as depression, encompassing a larger patient cohort and longer follow-up duration [19].

Our findings suggest that resolving POP and SUI may not uniformly enhance all aspects of mental health, demonstrating a clear positive impact primarily on anxiety traits. This observation implies that our patients may experience depression or insomnia that are not solely attributable to POP.

Given our prior observations linking psychiatric symptoms mainly to the subjective experience of POP and SUI [8], we opted to investigate not only changes in mental symptoms post-surgery but also the correlation between improvements in subjective symptoms of SUI and POP, such as LUTS, colorectal symptoms, or prolapse symptoms themselves, with changes in depression, anxiety, and insomnia questionnaires. Correlation analysis of specific problem severities revealed that improvements in prolapse symptoms themselves (assessed in POPDI 6) correlated with enhancements in both anxiety and depression. Likewise, enhancements in symptoms assessed by UDI 6 and CRADI 8 correlated with improvements in depression and anxiety scales. These findings suggest a beneficial effect of successful prolapse repair on mental well-being in these aspects.

Our analysis aligns with findings from other studies. For instance, research conducted by Ai et al. demonstrated that even conservative treatments, such as wearing a pessary for POP, improved the quality of life by alleviating prolapse symptoms and reducing depression symptoms in successfully treated patients [20]. Furthermore, studies by other researchers suggest that effective physiotherapy, which mitigates the subjective severity of pelvic floor disease symptoms, can also alleviate feelings of depression and anxiety [21]. Additionally, Pham et al. found that initial visits to urogynecological clinics, where patients receive information about their condition and treatment methods, can reduce anxiety in individuals with PFDs. This implies that addressing the underlying problem should further alleviate symptoms, as demonstrated in our study [22]. At the same time, Collin and colleagues showed that patients with high levels of anxiety did not have a worse subjective assessment of their health after pelvic organ prolapse surgery. This indirectly confirms our results showing that curing PFDs reduces anxiety rather than anxiety worsening the experience of PFDs [16]. Similar conclusions supporting our results were obtained by the authors assessing the lack of impact of generalized anxiety disorders on satisfaction with the use of pessary therapy in the case of prolapsus [23]. 

In the case of patients with SUI, effective surgical intervention led to a decrease in anxiety and insomnia severity, with a slight positive impact on depression severity. Concurrently, improvements in the assessment of quality of life, as indicated by the IIQ7 questionnaire, were associated with enhancements in depression and anxiety symptoms, suggesting a relationship between SUI and these mental health issues. Similar conclusions, albeit lacking empirical evidence from treated patients, were drawn in a study by Dermici et al., which relied solely on interviews and focused on a patient with SUI, OABs, and mixed urinary incontinence [24].

Large-scale Asian cohort studies have further substantiated the link between depression, anxiety, and SUI, identifying SUI as an independent risk factor for these mental health conditions and highlighting its association with significant work impairments [25]. These findings are consistent with the initial findings of our study published in 2023 [8], where we demonstrated the adverse impact of SUI by comparing depression and anxiety scales between patients with pelvic floor disorders and healthy individuals.

Notwithstanding the findings of previous researchers, it is pertinent to highlight that our team’s results constitute the first extensive investigations conducted on a large patient cohort, offering insights into the intervention impact following successful surgeries for both POP and SUI.

Regarding sleep disturbances, a study by Leng et al. demonstrated the co-occurrence of stress urinary incontinence and insomnia in affected patients, yet the causal relationship remains undetermined [26]. However, our findings, indicating a reduction in insomnia symptoms post-incontinence resolution, suggest that uncontrolled urine leakage may be a contributing factor to insomnia in patients with SUI.

The literature on sleep disorders in women suffering from POP is scarce. While our results revealed no correlation between reported symptoms in patients with POP and insomnia, Ghetti et al. reported a higher prevalence of sleep disturbances, such as difficulty falling asleep and interrupted sleep, in these patients compared to healthy individuals, mainly attributed to nocturia and pollakisuria [27]. Although our study focuses solely on insomnia, Ghetti’s work suggested a broader analysis of accompanying sleep symptoms.

In the current work, it was demonstrated that it is primarily the intensity of subjective symptoms and their perception that affect the mental state of patients, with a lesser impact on the objective assessment of the severity of SUI or POP. In addressing the importance of subjective symptoms in evaluating the mental health impact of pelvic floor disorders (PFDs), it is clear that patient-reported experiences offer valuable insights that go beyond what can be measured via objective clinical signs alone. Recognizing the intensity of a patient’s discomfort or pain and how they perceive their symptoms is crucial in understanding the full impact of PFDs on their mental well-being. However, this subjective aspect introduces complexity to the objective assessment of PFD severity. While clinical measures provide a standard way to quantify and track physical manifestations of PFDs, subjective symptoms are inherently personal and influenced by a range of psychological and social factors.

To balance these perspectives, a combined approach using both objective clinical assessments and patient-reported outcome measures (PROMs) should be considered. PROMs can bridge the gap between clinical evaluation and the patient’s lived experience, leading to more tailored and effective treatment plans. Ultimately, prioritizing a patient-centered approach that respects both the measurable aspects of PFDs and the personal experiences of those living with these conditions is essential for comprehensive care.

A potential mechanism through which PFDs affect the mental state of patients is the feeling of limitation in physical, professional, and social functioning, often accompanied by feelings of exclusion and stigmatization. Additionally, chronic fatigue associated with sleep disturbances and a deterioration in financial situation due to expenses on medications, medical care, and absorbent products contribute to this impact.

In summary, our current study, employing a before-and-after analysis of successful POP surgery, demonstrates a reduction in depression and anxiety post-treatment, with an additional improvement in insomnia in SUI cases. The rigorous intervention impact assessment design of our study underscores the significance of the results obtained and enables robust conclusions to be drawn. The results presented constitute a new, significant contribution to the existing knowledge on mental disorders in patients with PFDs. It has been confirmed that patients with PFDs often exhibit depressive changes, anxiety states, or sleep disorders. However, until now, their mutual relationship has not been unequivocally determined. It is unclear whether PFDs cause the disorders above or whether existing disorders intensify the symptoms of PFDs. By analyzing the mental state of patients before and after a successful surgery, we demonstrated a clear causal relationship in this area.

In many cases, pelvic floor disorders coexist, so it seems reasonable to undertake future research on patients with comorbidities and assess their impact on the mental health of the affected individuals. At the same time, the analysis of individual disease entities represents the greatest limitation of this study.

## 5. Conclusions

In summary, we propose a notable causal relationship between PFDs, specifically POP and SUI, and mental health conditions, particularly anxiety and depression.

The observed correlation between the alleviation of anxiety and depression severity and the improvement in PFD symptoms among successfully treated patients underscores the significant impact of POP and SUI on mental health issues in this demographic. This underscores the critical role of effective treatment for these conditions in enhancing patients’ quality of life, including their mental well-being. Furthermore, it suggests that addressing PFDs causally could potentially alleviate the burden on healthcare systems in managing anxiety and depression disorders.

## Figures and Tables

**Table 1:** Demographic characteristics of patients with POP (*n* = 187).

	Mean Value	Minimum	Maximum	SD
Age	61.5	33.0	87.0	11.7
Height	162.3	145.0	178.0	5.9
Weight	71.3	48.0	104.0	11.3
BMI	27.1	18.8	39.1	4.2
Number of deliveries	1.9	0.0	5.0	1.0
Number of vaginal deliveries	2.1	0.0	8.0	1.2
Number of cesarean sections	0.1	0.0	3.0	0.3
Age of first delivery	24.8	17.0	36.0	4.1
Age of last delivery	30.3	19.0	58.0	5.9

**Table 2:** Demographic characteristics of patients with SUI (*n* = 161).

	Mean	Minimum	Maximum	SD
Age	52.5	33	84	10.8
Height (cm)	164.9	150.0	180.0	6.1
Weight (kg)	75.0	47.0	116.0	13.8
BMI	27.5	19.1	40.2	4.6
Number of deliveries	1.91	0.00	5.00	1.03
Number of vaginal deliveries	1.7	0.0	5.0	1.1
Number of cesarean sections	0.19	0.00	3.00	0.54
Age of first delivery	25.5	16.0	41.0	5.1
Age of last delivery	29.8	17.0	47.0	5.7

**Table 3:** Psychiatric symptoms after successful POP surgery.

	Before Surgery	After Surgery	*p*
STAI	80.90 ± 16.06	74.25 ± 14.42	0.0002
CESD	29.32 ± 7.32	29.73 ± 6.66	0.63
ISI	8.26 ± 5.41	7.56 ± 5.87	0.21

**Table 4:** Psychiatric symptoms after successful SUI surgery.

	Before Surgery	After Surgery	*p*
STAI	87.51 ± 14.20	76.88 ± 13.85	0.000001
CESD	32.24 ± 8.98	30.58 ± 7.67	0.12
ISI	11.10 ± 5.15	9.24 ± 5.54	0.0009

## Data Availability

Data available on request.
